# Supplementary material for: Systemic and Mucosal Antibody Correlates of Protection Against Bordetella pertussis in a Controlled Human Infection Model
Source: medRxiv. 2026 Jun 23:2026.06.18.26355881. Preprint. [Version 1] doi: 10.64898/2026.06.18.26355881 (PMC13321178; doi:10.64898/2026.06.18.26355881)

medRxiv preprint doi: https://doi.org/10.64898/2026.06.18.26355881; this version posted June 23, 2026. The copyright holder has placed this preprint (which was not certified by peer review) in the Public Domain. It is no longer restricted by copyright. Anyone can legally share, reuse, remix, or adapt this material for any purpose without crediting the original authors.

Figure S1. Antibody profiles of wP vaccinees. (a-b) Heatmap demonstrating log2 fold changes in aP-antigen specific antibody features (AUC, day -1 to 3) between infected and non-infected participants in serum (a) and nasal wash (b). Color intensity reflects the direction and magnitude of enrichment, with red indicating features elevated in infected individuals and blue indicating features enriched in non-infected individuals. Features not examined are crossed. Significances were assessed using Mann–Whitney U tests and corrected for multiple testing using the Benjamini–Hochberg procedure. Asterisks indicate adjusted P values with *P < 0.05, **P < 0.01, ***P < 0.001

Figure S2. Serum antibody correlates of B. pertussis symptomology in aP-vaccinees. (a) Volcano plot showing pairwise comparison depicting serum antibody features between aP vaccinated asymptomatic (purple) and symptomatic (yellow) participants following controlled human infection. Features are plotted as $\log_2$ fold change (dose-corrected AUC day 1 to 7) versus $-\log_{10}$ (p value). The horizontal dashed line indicates p = 0.05, and the vertical dashed line denotes the manually selected significant threshold. **(b)** Partial least squares discriminant analysis (PLS-DA) scores plot demonstrating separation between asymptomatic (purple) or symptomatic (yellow) participants based on LASSO-selected serum antibody profiles (dose-corrected AUC day 1 to 7). Each dot represents an individual participant; the number of individuals (n) is indicated; ellipses denote 75% confidence regions assuming a multivariate t distribution. Marginal density plots for Latent Variables- LV1 and LV2 are shown. **(c)** Directed variable importance in projection (VIP) scores highlighting top discriminatory serum features (VIP > 1.0) enriched in asymptomatic (purple) or symptomatic (yellow) individuals. **(d)** Boxplots representing medians, interquartile ranges [IQRs], minima, and maxima of representative discriminative features (VIP >1.0, panel c) comparing asymptomatic (purple) or symptomatic (yellow) individuals; the number of individuals is the same as in panel **(b)**. The measurements were dose-corrected and z-scored for AUC day 1 to 7. Significances were assessed using Mann–Whitney U tests and corrected for multiple testing using the Benjamini–Hochberg procedure. Asterisks indicate adjusted P values with *P < 0.05, **P < 0.01, ***P < 0.001. **(e)** The model performance was validated using permutation tests. Classification accuracy is shown for models built using selected features (panel c), random features, or permuted labels. Violin plots show the distribution from 100 repetitions for each model type. P values represent the median exact permutation-derived P value across repetitions. Significances were assessed using Mann–Whitney U tests and corrected for multiple testing using the Benjamini–Hochberg procedure. Asterisks indicate adjusted P values with *P < 0.05, **P < 0.01, ***P < 0.001. **(f)** Radar/Polar plots depicting mean percentile ranks of antigen-specific antibody feature distributions for representative discriminative antigens (VIP >1.0, panel c) in non-infected (top) versus infected (bottom) individuals. Percentile rank scores were determined for each antibody feature across all individuals.

Figure S3. Serum antibody correlates of B. pertussis symptomology in wP-vaccinees. (a) Volcano plot showing pairwise comparison depicting serum antibody features between wP vaccinated asymptomatic (purple) and symptomatic (yellow) participants following controlled human infection. Features are plotted as $\log_2$ fold change (dose-corrected AUC day 1 to 7) versus $-\log_{10}$ (p value). The horizontal dashed line indicates p = 0.05, and the vertical dashed line denotes the manually selected

medRxiv preprint doi: https://doi.org/10.64898/2026.06.18.26355881; this version posted June 23, 2026. The copyright holder has placed this preprint (which was not certified by peer review) in the Public Domain. It is no longer restricted by copyright. Anyone can legally share, reuse, remix, or adapt this material for any purpose without crediting the original authors.

significant threshold. **(b)** PLS-DA scores plot demonstrating separation between asymptomatic (purple) or symptomatic (yellow) participants based on LASSO-selected serum antibody profiles (dose-corrected AUC day 1 to 7). Each dot represents an individual participant; the number of individuals (n) is indicated; ellipses denote 75% confidence regions assuming a multivariate t distribution. Marginal density plots for LV1 and LV2 are shown. **(c)** Directed VIP scores highlighting top discriminatory serum features (VIP > 1.0) enriched in asymptomatic (purple) or symptomatic (yellow) individuals. **(d)** Boxplots representing medians, IQRs, minima, and maxima of representative discriminative features (VIP >1.0, panel c) comparing asymptomatic (purple) or symptomatic (yellow) individuals; the number of individuals is the same as in panel **(b)**. The measurements were dose-corrected and z-scored for AUC day 1 to 7. Significances were assessed using Mann–Whitney U tests and corrected for multiple testing using the Benjamini–Hochberg procedure. Asterisks indicate adjusted P values with $*P < 0.05$, $**P < 0.01$, $***P < 0.001$. **(e)** The model performance was validated using permutation tests. Classification accuracy is shown for models built using selected features (panel c), random features, or permuted labels. Violin plots show the distribution from 100 repetitions for each model type. P values represent the median exact permutation-derived P value across repetitions. Significances were assessed using Mann–Whitney U tests and corrected for multiple testing using the Benjamini–Hochberg procedure. Asterisks indicate adjusted P values with $*P < 0.05$, $**P < 0.01$, $***P < 0.001$. **(f)** Radar/Polar plots depicting mean percentile ranks of antigen-specific antibody feature distributions for representative discriminative antigens (VIP >1.0, panel c) in non-infected (top) versus infected (bottom) individuals. Percentile rank scores were determined for each antibody feature across all individuals.

Figure S4. Mucosal antibody correlates of B. pertussis symptomology in aP-vaccinees. (a) Volcano plot showing pairwise comparison depicting nasal wash antibody features between aP vaccinated asymptomatic (purple) and symptomatic (yellow) participants following controlled human infection. Features are plotted as $\log_2$ fold change (dose-corrected AUC day 1 to 7) versus $-\log_{10}$ (p value). The horizontal dashed line indicates $p = 0.05$, and the vertical dashed line denotes the manually selected significant threshold. **(b)** PLS-DA scores plot demonstrating separation between asymptomatic (purple) or symptomatic (yellow) participants based on LASSO-selected nasal wash antibody profiles (dose-corrected AUC day 1 to 7). Each dot represents an individual participant; the number of individuals (n) is indicated; ellipses denote 75% confidence regions assuming a multivariate t distribution. Marginal density plots for LV1 and LV2 are shown. **(c)** VIP scores highlighting top discriminatory nasal wash features (VIP > 1.0) enriched in asymptomatic (purple) or symptomatic (yellow) individuals. **(d)** Boxplots representing medians, IQRs, minima, and maxima of representative discriminative features (VIP >1.0, panel c) comparing asymptomatic (purple) or symptomatic (yellow) individuals; the number of individuals is the same as in panel **(b)**. The measurements were dose-corrected and z-scored for AUC day 1 to 7. Significances were assessed using Mann–Whitney U tests and corrected for multiple testing using the Benjamini–Hochberg procedure. Asterisks indicate adjusted P values with $*P < 0.05$, $**P < 0.01$, $***P < 0.001$. **(e)** The model performance was validated using permutation tests. Classification accuracy is shown for models built using selected features (panel c), random features, or permuted labels. Violin plots show the distribution from 100 repetitions for each model type. P values represent the median exact permutation-derived P value across repetitions. Significances were assessed using Mann–Whitney U tests and corrected for multiple testing using the Benjamini–Hochberg procedure. Asterisks indicate adjusted P values with $*P < 0.05$, $**P < 0.01$, $***P < 0.001$. **(f)** Radar/Polar plots depicting mean percentile ranks of antigen-specific antibody feature distributions for representative discriminative antigens (VIP >1.0, panel c) in non-infected (top) versus infected (bottom) individuals. Percentile rank scores were determined for each antibody feature across all individuals.

Figure S5. Mucosal antibody correlates of B. pertussis symptomology in wP-vaccinees. (a) Volcano plot showing pairwise comparison depicting nasal wash antibody features between wP vaccinated

medRxiv preprint doi: https://doi.org/10.64898/2026.06.18.26355881; this version posted June 23, 2026. The copyright holder has placed this preprint (which was not certified by peer review) in the Public Domain. It is no longer restricted by copyright. Anyone can legally share, reuse, remix, or adapt this material for any purpose without crediting the original authors.

asymptomatic (purple) and symptomatic (yellow) participants following controlled human infection. Features are plotted as $\log_2$ fold change (dose-corrected AUC day 1 to 7) versus $-\log_{10}$ (p value). The horizontal dashed line indicates p = 0.05, and the vertical dashed line denotes the manually selected significant threshold. **(b)** PLS-DA scores plot demonstrating separation between asymptomatic (purple) or symptomatic (yellow) participants based on LASSO-selected nasal wash antibody profiles (dose-corrected AUC day 1 to 7). Each dot represents an individual participan; the number of individuals (n) is indicated t; ellipses denote 75% confidence regions assuming a multivariate t distribution. Marginal density plots for LV1 and LV2 are shown. **(c)** VIP scores highlighting top discriminatory nasal wash features (VIP > 1.0) enriched in asymptomatic (purple) or symptomatic (yellow) individuals. **(d)** Boxplots representing medians, IQRs, minima, and maxima of representative discriminative features (VIP >1.0, panel c) comparing asymptomatic (purple) or symptomatic (yellow) individuals; the number of individuals is the same as in panel **(b)**. The measurements were dose-corrected and z-scored for AUC day 1 to 7. Significances were assessed using Mann–Whitney U tests and corrected for multiple testing using the Benjamini–Hochberg procedure. Asterisks indicate adjusted P values with $*P < 0.05$, $**P < 0.01$, $***P < 0.001$. **(e)** The model performance was validated using permutation tests. Classification accuracy is shown for models built using selected features (panel c), random features, or permuted labels. Violin plots show the distribution from 100 repetitions for each model type. P values represent the median exact permutation-derived P value across repetitions. Significances were assessed using Mann–Whitney U tests and corrected for multiple testing using the Benjamini–Hochberg procedure. Asterisks indicate adjusted P values with $*P < 0.05$, $**P < 0.01$, $***P < 0.001$. **(f)** Radar/Polar plots depicting mean percentile ranks of antigen-specific antibody feature distributions for representative discriminative antigens (VIP >1.0, panel c) in non-infected (top) versus infected (bottom) individuals. Percentile rank scores were determined for each antibody feature across all individuals.

Figure S6. Serum and mucosal antibody correlates of B. pertussis symptomology in aP-vaccinees. (a) Volcano plot showing pairwise comparison depicting serum and nasal wash antibody features between aP vaccinated asymptomatic (purple) and symptomatic (yellow) participants following controlled human infection. Features are plotted as $\log_2$ fold change (dose-corrected AUC day 1 to 7) versus $-\log_{10}$ (p value). The horizontal dashed line indicates p = 0.05, and the vertical dashed line denotes the manually selected significant threshold. **(b)** PLS-DA scores plot demonstrating separation between asymptomatic (purple) or symptomatic (yellow) participants based on LASSO-selected serum and nasal wash antibody profiles (dose-corrected AUC day 1 to 7). Each dot represents an individual participant; the number of individuals (n) is indicated; ellipses denote 75% confidence regions assuming a multivariate t distribution. Marginal density plots for LV1 and LV2 are shown. **(c)** VIP scores highlighting top discriminatory antibody features (VIP > 1.0) enriched in asymptomatic (purple) or symptomatic (yellow) individuals. **(d)** Boxplots representing medians, IQRs, minima, and maxima of representative discriminative features (VIP >1.0, panel c) comparing asymptomatic (purple) or symptomatic (yellow) individuals; the number of individuals is the same as in panel **(b)**. The measurements were dose-corrected and z-scored for AUC day 1 to 7. Significances were assessed using Mann–Whitney U tests and corrected for multiple testing using the Benjamini–Hochberg procedure. Asterisks indicate adjusted P values with $*P < 0.05$, $**P < 0.01$, $***P < 0.001$. **(e)** The model performance was validated using permutation tests. Classification accuracy is shown for models built using selected features (panel c), random features, or permuted labels. Violin plots show the distribution from 100 repetitions for each model type. P values represent the median exact permutation-derived P value across repetitions. Significances were assessed using Mann–Whitney U tests and corrected for multiple testing using the Benjamini–Hochberg procedure. Asterisks indicate adjusted P values with $*P < 0.05$, $**P < 0.01$, $***P < 0.001$.

medRxiv preprint doi: https://doi.org/10.64898/2026.06.18.26355881; this version posted June 23, 2026. The copyright holder has placed this preprint (which was not certified by peer review) in the Public Domain. It is no longer restricted by copyright. Anyone can legally share, reuse, remix, or adapt this material for any purpose without crediting the original authors.

Figure S7. Serum and mucosal antibody correlates of B. pertussis symptomology in wP-vaccinees. **(a)** Volcano plot showing pairwise comparison depicting serum and nasal wash antibody features between wP vaccinated asymptomatic (purple) and symptomatic (yellow) participants following controlled human infection. Features are plotted as $\log_2$ fold change (dose-corrected AUC day 1 to 7) versus $-\log_{10}$ (p value). The horizontal dashed line indicates p = 0.05, and the vertical dashed line denotes the manually selected significant threshold. **(b)** PLS-DA scores plot demonstrating separation between asymptomatic (purple) or symptomatic (yellow) participants based on LASSO-selected serum and nasal wash antibody profiles (dose-corrected AUC day 1 to 7). Each dot represents an individual participant; the number of individuals (n) is indicated; ellipses denote 75% confidence regions assuming a multivariate t distribution. Marginal density plots for LV1 and LV2 are shown. **(c)** VIP scores highlighting top discriminatory antibody features (VIP > 1.0) enriched in asymptomatic (purple) or symptomatic (yellow) individuals. **(d)** Boxplots representing medians, IQRs, minima, and maxima of representative discriminative features (VIP >1.0, panel c) comparing asymptomatic (purple) or symptomatic (yellow) individuals; the number of individuals is the same as in panel **(b)**. The measurements were dose-corrected and z-scored for AUC day 1 to 7. Significances were assessed using Mann–Whitney U tests and corrected for multiple testing using the Benjamini–Hochberg procedure. Asterisks indicate adjusted P values with $*P < 0.05$, $**P < 0.01$, $***P < 0.001$. **(e)** The model performance was validated using permutation tests. Classification accuracy is shown for models built using selected features (panel c), random features, or permuted labels. Violin plots show the distribution from 100 repetitions for each model type. P values represent the median exact permutation-derived P value across repetitions. Significances were assessed using Mann–Whitney U tests and corrected for multiple testing using the Benjamini–Hochberg procedure. Asterisks indicate adjusted P values with $*P < 0.05$, $**P < 0.01$, $***P < 0.001$. **(f)** Radar/Polar plots depicting mean percentile ranks of antigen-specific antibody feature distributions for representative discriminative antigens (VIP >1.0, panel c) in non-infected (top) versus infected (bottom) individuals. Percentile rank scores were determined for each antibody feature across all individuals.

Figure S8. Integrated serum and mucosal antibody features improve discrimination of symptomatic *B. pertussis* cases in wP- and aP- vaccinees. (a) UpSet plot showing the overlap of discriminative antigen sets derived from LASSO/PLSDA models using antibody features in serum, nasal wash, or combined serum + nasal wash in wP- and aP- vaccinated participants. (b,d) Receiver operating characteristic (ROC) curves comparing model performance for classifying asymptomatic versus symptomatic participants using serum alone, nasal wash alone, or combined serum + nasal wash antibody features across antigen classes (aP vaccine antigens, LASSO-selected antigens, and microbiome antigens) in aP-vaccinated (b) and wP-vaccinated (e) participants. (c,e) Bar plot summarizing ROC–AUC values for each model in aP-vaccinated (c) and wP-vaccinated (e) participants. Bars represent mean ROC–AUC ± S.D. (d,g).

Supplemental Table

Table S1. Metadata and antigen panel of systems serology. PBS subtracted values for distinct antigen-specific antibody features.

Table S2. Features enriched in various categories as identified by univariate analysis (volcano plots)

Table S3. Serum correlates of protection from infection and symptomatology identified by LASSO-PLSDA

Table S4. Nasal wash correlates of protection from infection and symptomatology identified by LASSO-PLSDA

Table S5. Combined serum and nasal wash correlates of protection from infection and symptomatology identified by LASSO-PLSDA

Table S6. Non-infected VIP features identified across sample types in wP-vaccinated participants

medRxiv preprint doi: https://doi.org/10.64898/2026.06.18.26355881; this version posted June 23, 2026. The copyright holder has placed this preprint (which was not certified by peer review) in the Public Domain. It is no longer restricted by copyright. Anyone can legally share, reuse, remix, or adapt this material for any purpose without crediting the original authors.

Table S7. Asymptomatic VIP features identified across sample types in aP- and wP-vaccinated participants

Figure S1

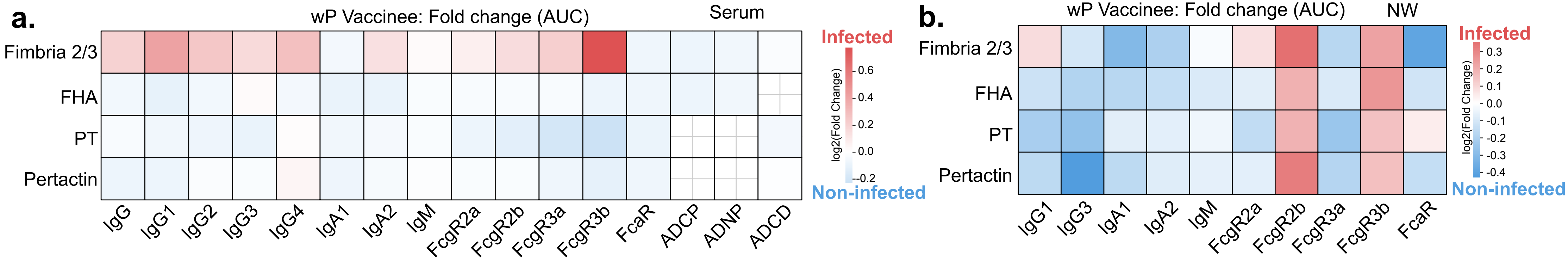
a.
wP Vaccinee: Fold change (AUC)
Serum
Fimbria 2/3
FHA
PT
Pertactin
IgG
IgG1
IgG2
IgG3
IgG4
IgA1
IgA2
IgM
FcgR2a
FcgR2b
FcgR3a
FcgR3b
FcaR
ADCP
ADNP
ADCD
Infected
Non-infected
log2(Fold Change)
0.6
0.4
0.2
0.0
-0.2
b.
wP Vaccinee: Fold change (AUC)
NW
Fimbria 2/3
FHA
PT
Pertactin
IgG1
IgG3
IgA1
IgA2
IgM
FcgR2a
FcgR2b
FcgR3a
FcgR3b
FcaR
Infected
Non-infected
log2(Fold Change)
0.3
0.2
0.1
0.0
-0.1
-0.2
-0.3
-0.4

Figure S2

medRxiv preprint doi: https://doi.org/10.64898/2026.06.18.26355881; this version posted June 23, 2026. The copyright holder has placed this preprint (which was not certified by peer review) in the Public Domain. It is no longer restricted by copyright. Anyone can legally share, reuse, remix, or adapt this material for any purpose without crediting the original authors.

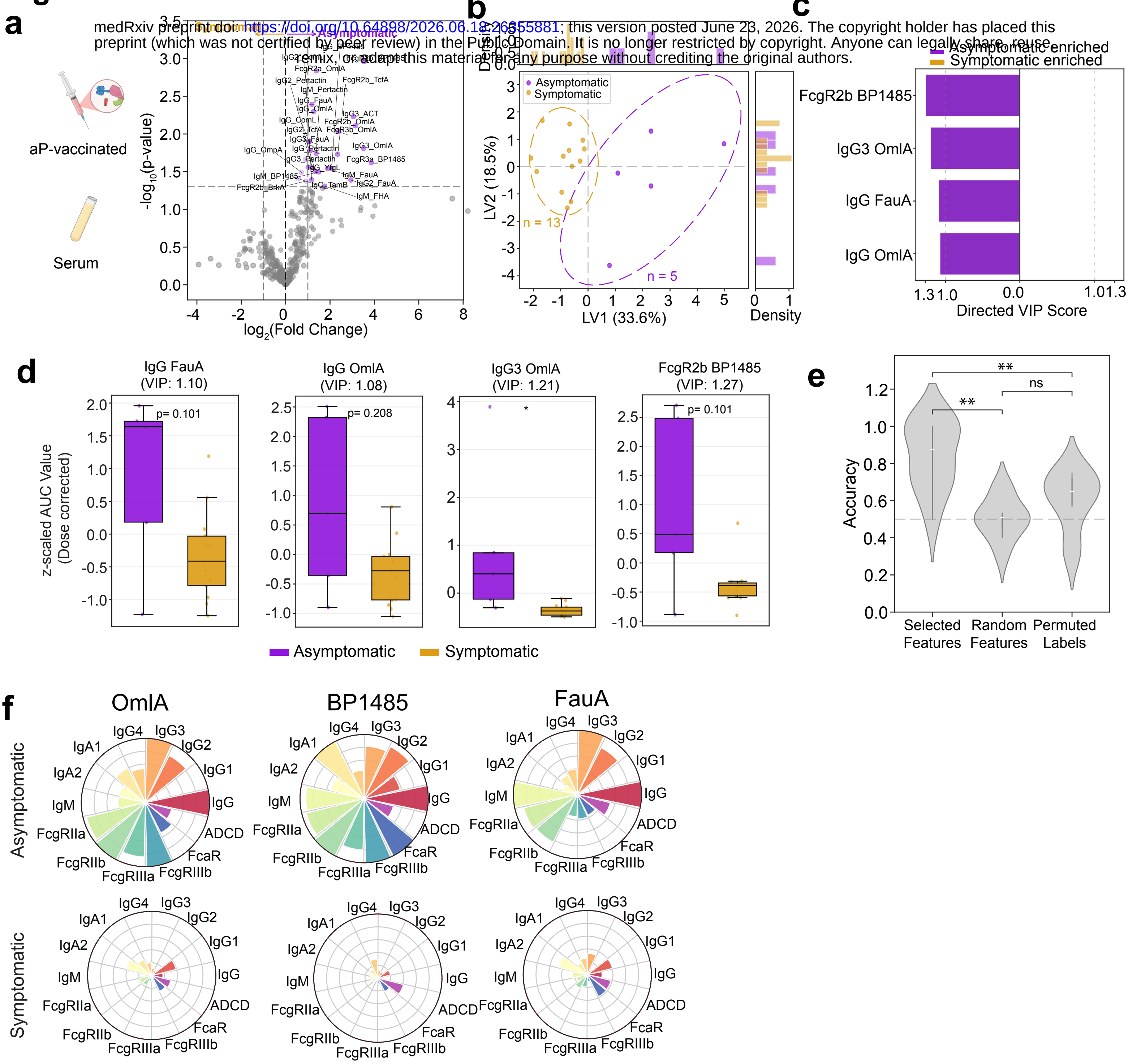

Figure S3

medRxiv preprint doi: https://doi.org/10.64898/2026.06.18.26355881; this version posted June 23, 2026. The copyright holder has placed this preprint (which was not certified by peer review) in the Public Domain. It is no longer restricted by copyright. Anyone can legally share, reuse, remix, or adapt this material for any purpose without crediting the original authors.

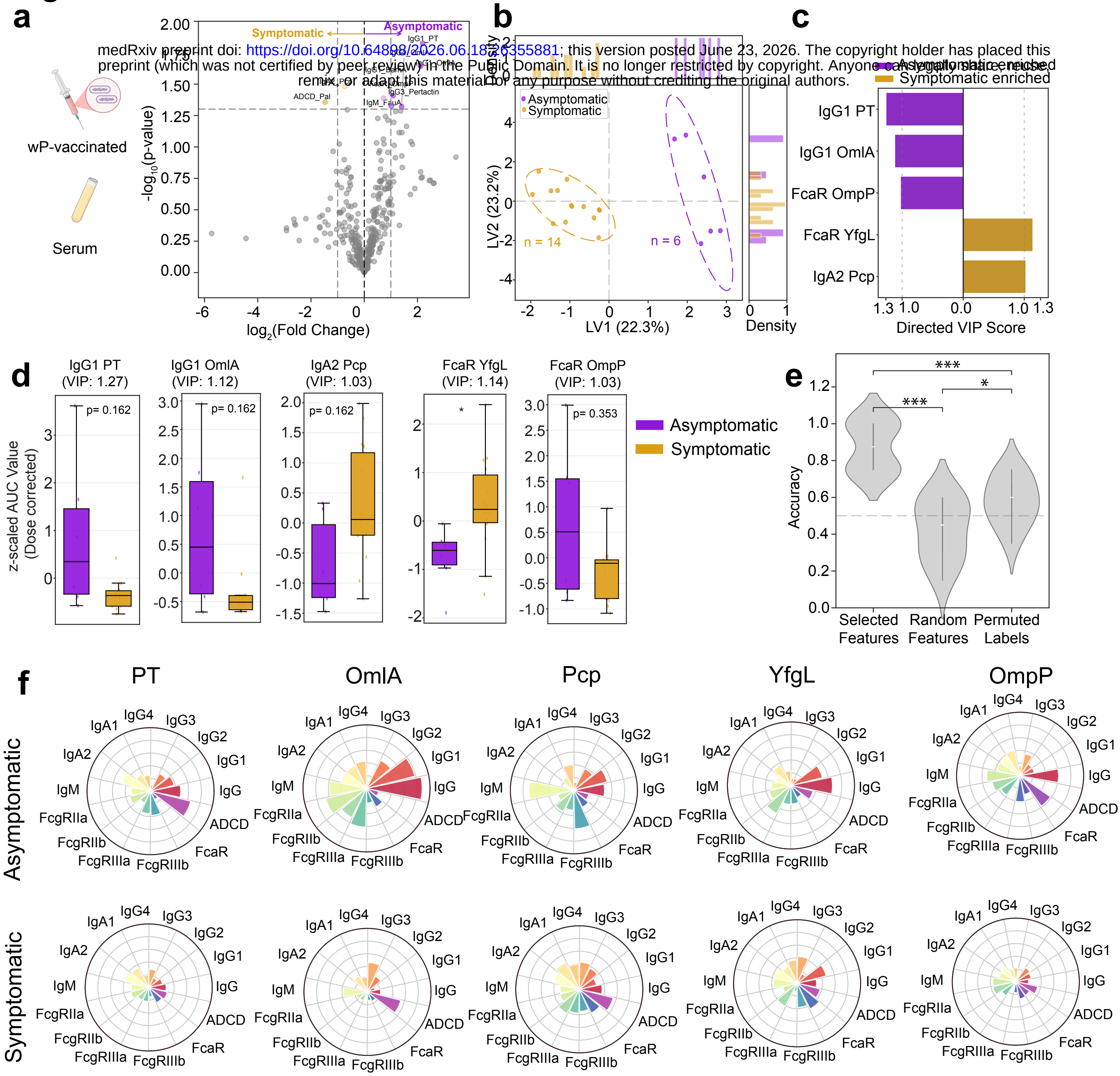

Figure S4

medRxiv preprint doi: https://doi.org/10.64898/2026.06.18.26355881; this version posted June 23, 2026. The copyright holder has placed this preprint (which was not certified by peer review) in the Public Domain. It is no longer restricted by copyright. Anyone can legally share, reuse, remix, or adapt this material for any purpose without crediting the original authors.

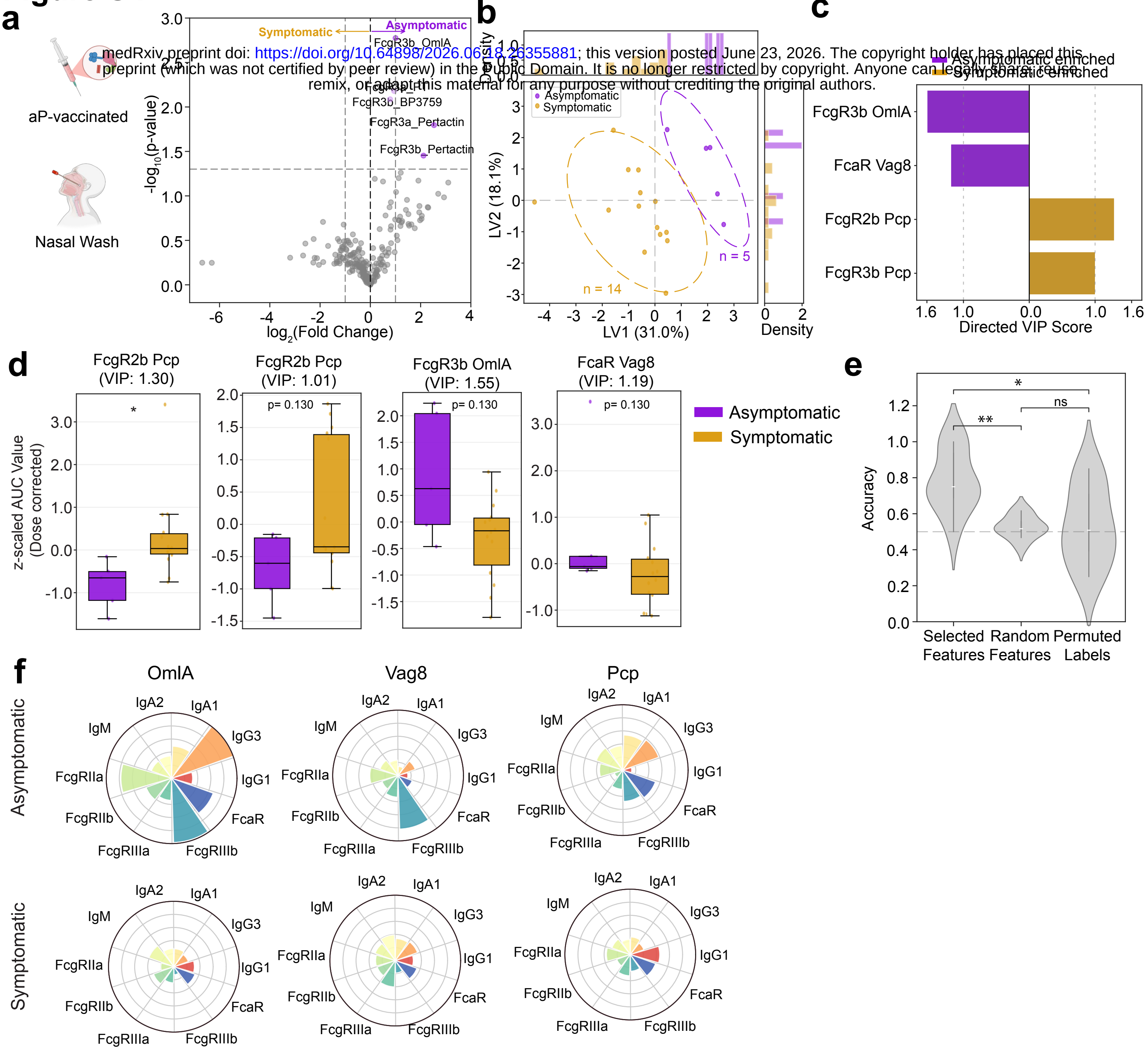

Figure S5

medRxiv preprint doi: https://doi.org/10.64898/2026.06.18.26355881; this version posted June 23, 2026. The copyright holder has placed this preprint (which was not certified by peer review) in the Public Domain. It is no longer restricted by copyright. Anyone can legally share, reuse, remix, or adapt this material for any purpose without crediting the original authors.

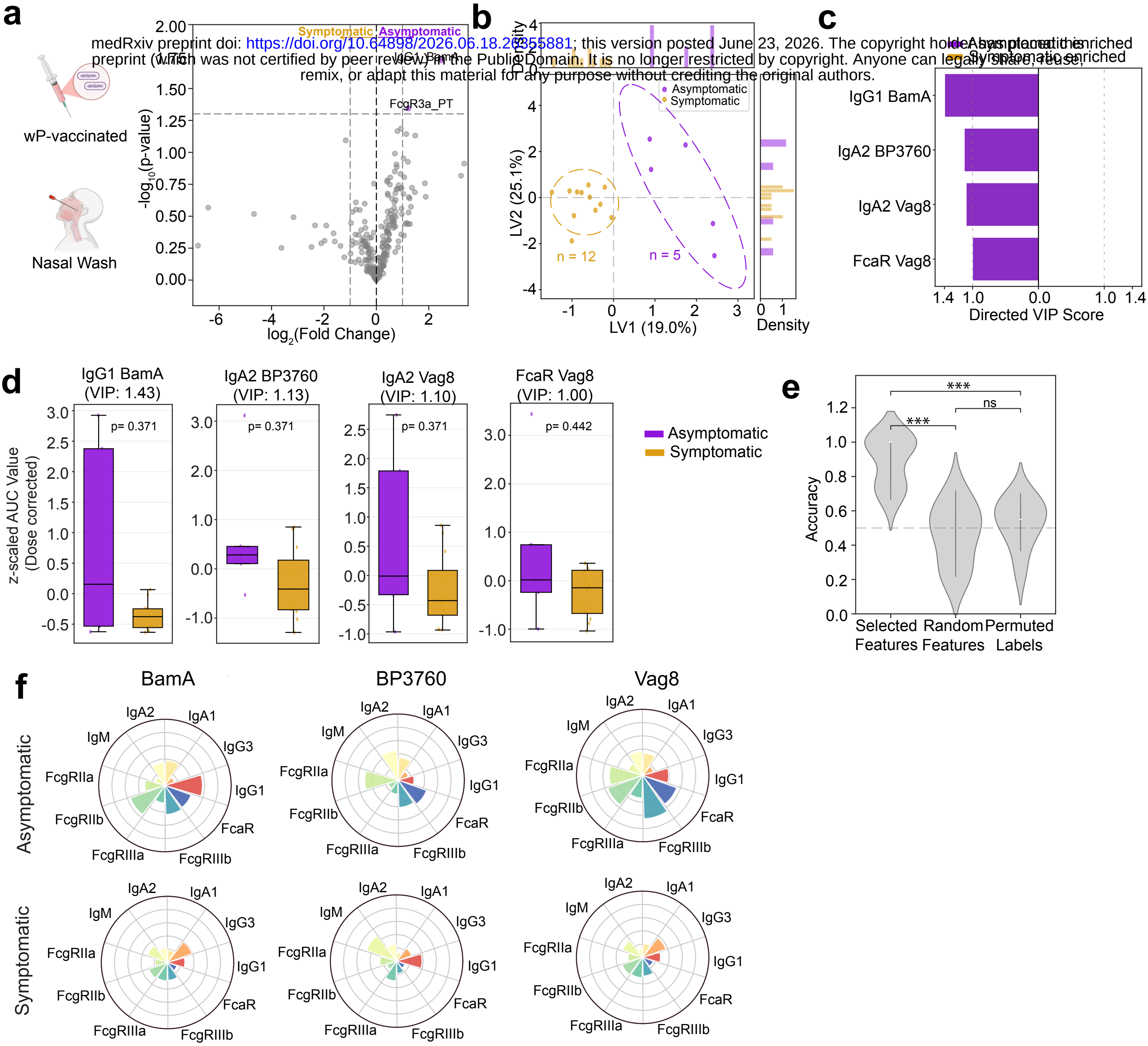

Figure S6

medRxiv preprint doi: https://doi.org/10.64898/2026.05.18.26355881; this version posted June 23, 2026. The copyright holder has placed this preprint (which was not certified by peer review) in the Public Domain. It is no longer restricted by copyright. Anyone can legally share, reuse, remix, or adapt this material for any purpose without crediting the original authors.

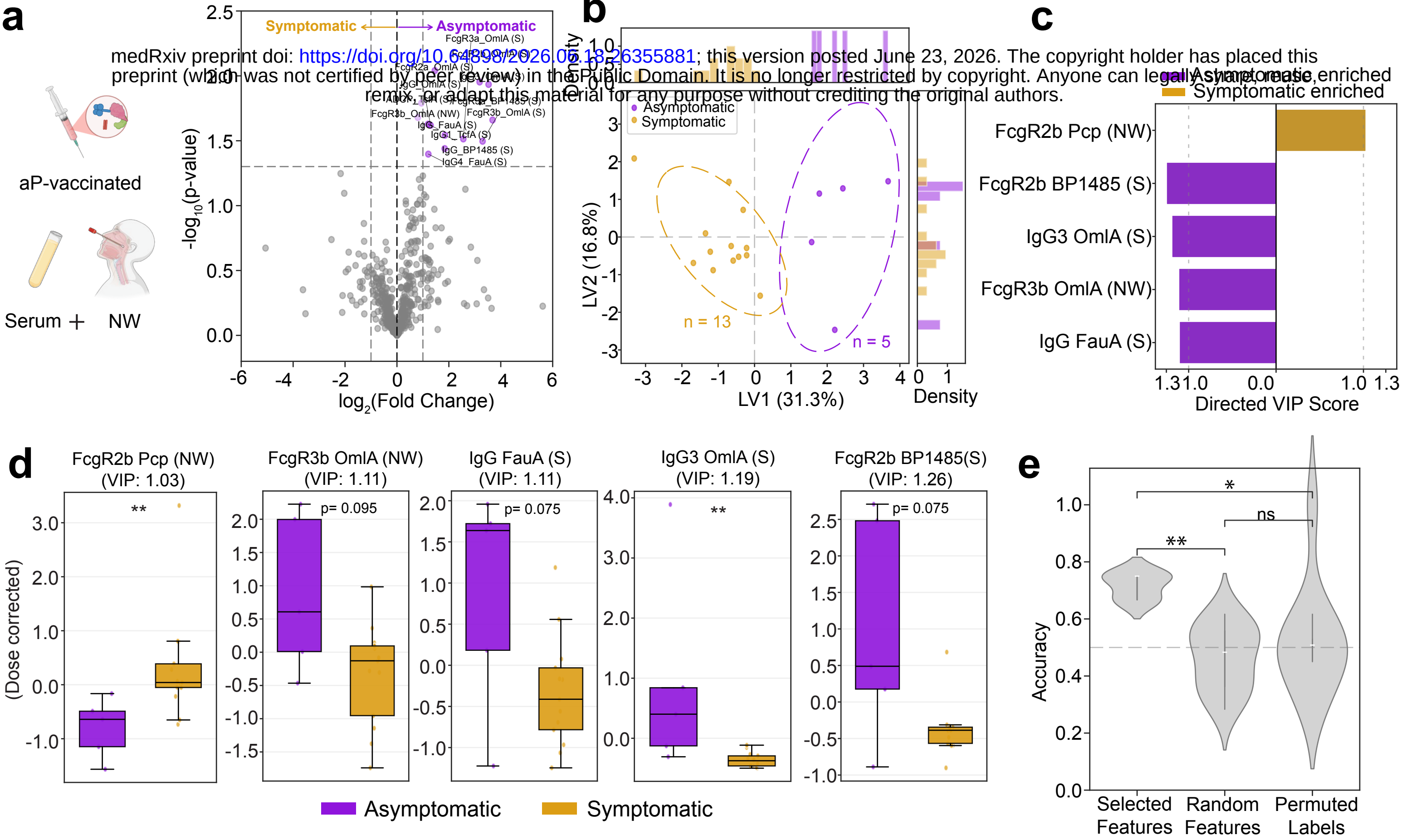

Figure S7

medRxiv preprint doi: https://doi.org/10.64898/2026.06.18.26355881; this version posted June 23, 2026. The copyright holder has placed this preprint (which was not certified by peer review) in the Public Domain. It is no longer restricted by copyright. Anyone can legally share, reuse, remix, or adapt this material for any purpose without crediting the original authors.

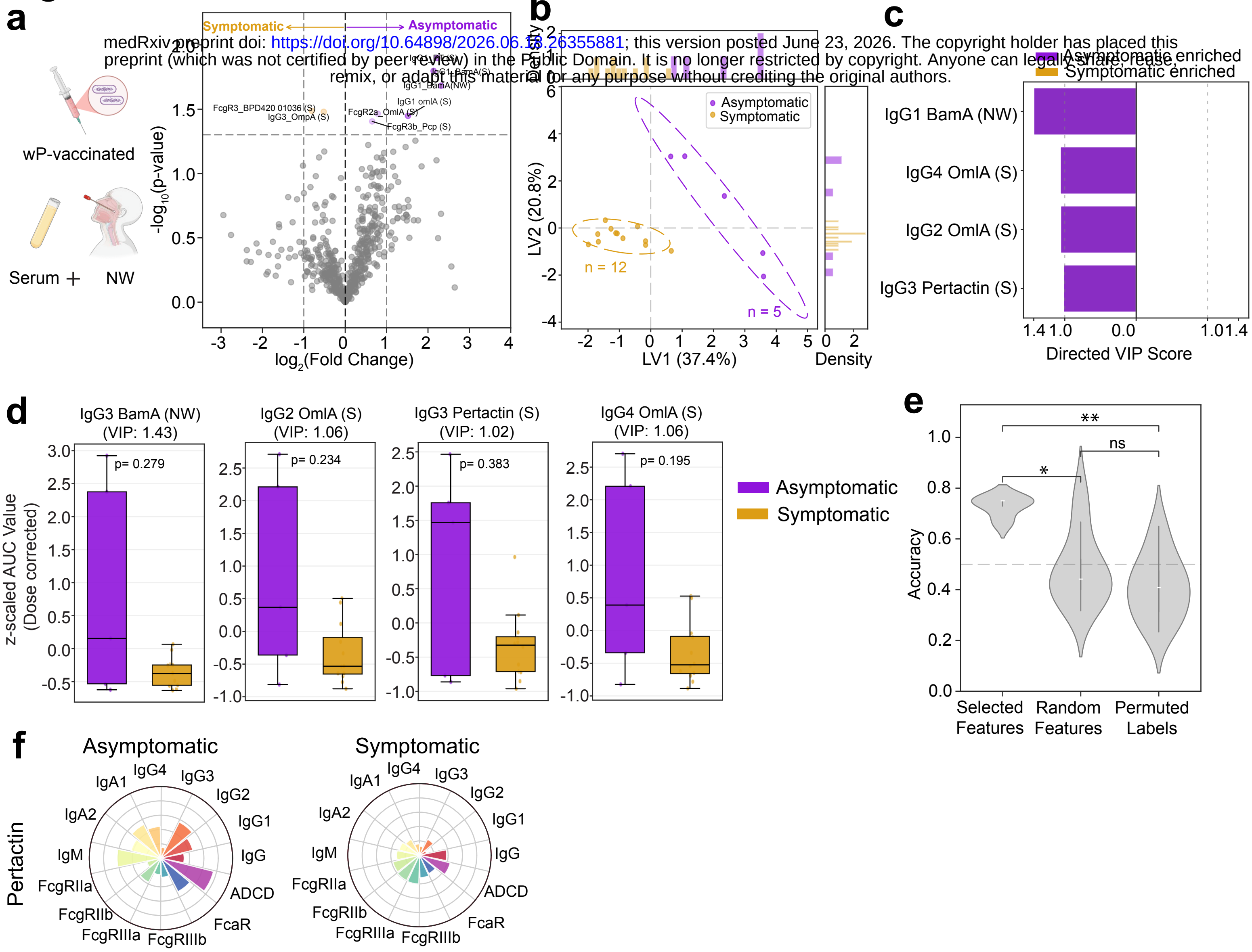

Figure S8

medRxiv preprint doi: https://doi.org/10.64898/2026.06.18.26355881; this version posted June 23, 2026. The copyright holder has placed this preprint (which was not certified by peer review) in the Public Domain. It is no longer restricted by copyright. Anyone can legally share, reuse, remix, or adapt this material for any purpose without crediting the original authors.

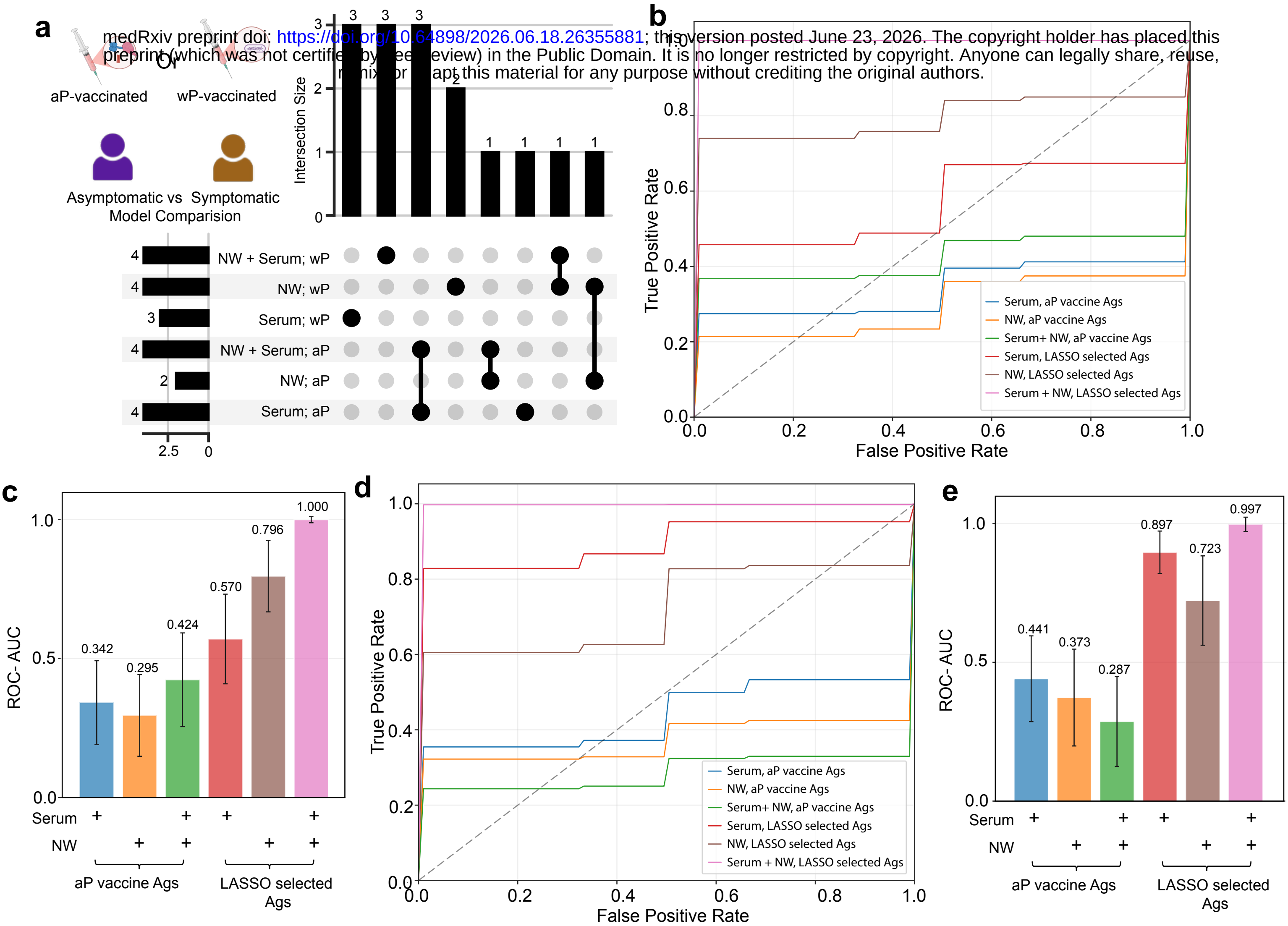

Supplement: Supplementary file 1 [file NIHPP2026.06.18.26355881V1-supplement-1.pdf]
